# On rare variants in principal component analysis of population stratification

**DOI:** 10.1186/s12863-020-0833-x

**Published:** 2020-03-17

**Authors:** Shengqing Ma, Gang Shi

**Affiliations:** State Key Laboratory of Integrated Services Networks, Xidian University, 2 South Taibai Road, Xi’an, 710071 Shaanxi China

**Keywords:** Rare variant, Population stratification, Principal component analysis, Single nucleotide polymorphism

## Abstract

**Background:**

Population stratification is a known confounder of genome-wide association studies, as it can lead to false positive results. Principal component analysis (PCA) method is widely applied in the analysis of population structure with common variants. However, it is still unclear about the analysis performance when rare variants are used.

**Results:**

We derive a mathematical expectation of the genetic relationship matrix. Variance and covariance elements of the expected matrix depend explicitly on allele frequencies of the genetic markers used in the PCA analysis. We show that inter-population variance is solely contained in *K* principal components (PCs) and mostly in the largest *K*-1 PCs, where *K* is the number of populations in the samples. We propose F_PC_, ratio of the inter-population variance to the intra-population variance in the *K* population informative PCs, and *d*^2^, sum of squared distances among populations, as measures of population divergence. We show analytically that when allele frequencies become small, the ratio F_PC_ abates, the population distance *d*^2^ decreases, and portion of variance explained by the *K* PCs diminishes. The results are validated in the analysis of the 1000 Genomes Project data. The ratio F_PC_ is 93.85, population distance *d*^2^ is 444.38, and variance explained by the largest five PCs is 17.09% when using with common variants with allele frequencies between 0.4 and 0.5. However, the ratio, distance and percentage decrease to 1.83, 17.83 and 0.74%, respectively, with rare variants of frequencies between 0.0001 and 0.01.

**Conclusions:**

The PCA of population stratification performs worse with rare variants than with common ones. It is necessary to restrict the selection to only the common variants when analyzing population stratification with sequencing data.

## Background

Genome-wide association studies (GWAS) [1] have identified a considerable number of sequence variants, such as single nucleotide polymorphisms (SNPs), associated with human diseases or traits. Population stratification—allele frequencies of genetic markers of the studied samples having significant differences owing to systematic ancestry differences—can cause false positive results in case-control as well as cohort studies [2, 3]. Association mapping based on rare variants are much more susceptible to subtle effects of population stratification and therefore, more likely to yield false positive results [4]. From a population genetics point of view, exploring population structure is important for understanding the evolutionary history of populations. Many methods and software are available to study the population stratification, such as the principal component analysis (PCA) implemented in EIGENSOFT [5, 6], the multidimensional scaling analysis in PLINK [7], the clustering analysis in STRUCTURE [8, 9], and fastSTRUCTURE [10]. Recently, controlling population stratification in the association analysis using linear mixed models [11–14] was also suggested. Based on a large number of common variants whose minor allele frequencies (MAFs) are larger than 5%, the PCA of population structure is widely applied in GWAS.

With the advance of high-throughput sequencing technology, as well as the enormous reduction of the cost, it is capable and affordable in genetic studies to detect additional low-frequency and rare variants (MAF < 1%) [15]. Furthermore, existing sequencing data suggest that the vast majority of rare variants are population-specific. In the 1000 Genomes Project data [16, 17], there are a total of 77 million biallelic SNPs, among which 65 million are rare and 52 million are polymorphic in one of the five continental ancestry populations: East Asian (EAS), South Asian (SAS), African (AFR), European (EUR), American (AMR). It seems that rare variants are more informative in distinguishing population structure than common ones. However, the efficacy of using rare variants in population stratification analysis remains controversial [18–21].

A number of efforts have been made concerning the use of low-frequency and rare variants in population stratification analysis. Baye et al. illustrated that more fine substructure can be detected using rare variants and suggested that more SNPs are required to account for a similar level of population structure using rare variants compared to common ones [18]. Siu et al. showed that rare variants have a much higher power to identify population substructure than common variants [19]. In contrast, Zhang et al. demonstrated that PCAs based on common and less-frequency SNPs perform better than those based on rare ones in separating European and African samples [20]. The authors further concluded that there is little added value for PCA of population stratification with rare variants only [21]. All existing work was based on analysis of genotype data from the 1000 Genomes Project with known population structure.

In this work, we investigate how rare variants affect PCA of population stratification from a theoretical perspective. We derive mathematical expectation of the genetic relationship matrix (GRM) [22]. The GRM is commonly computed from the observed genotypes and eigen-decomposed in the analysis of population stratification. Elements of the expected genetic relationship matrix (EGRM), however, depend explicitly on the allele frequencies of the markers used. We show that inter-population variance is solely contained in *K* principal components (PCs) and mostly in the largest *K*-1 PCs, where *K* is the number of populations in the sample. We propose F_PC_, ratio of the inter-population variance to the intra-population variance in the *K* population informative PCs, and *d*^2^, sum of squared distances among populations, as measures of population divergence. We show analytically that when allele frequencies become small, the ratio F_PC_ abates, the population distance *d*^2^ decreases, and portion of variance explained by the *K* PCs diminishes. Therefore, the PCA of population stratification performs worse with rare variants than with common ones. The results are further validated in the analysis of the 1000 Genomes Project data with 2504 individuals from five continental populations.

## Methods

### Genetic relationship matrix

In the scenario where genotype data of individuals is sampled from *K* populations, there are *N*_*k*_ individuals in population *k* and the number of individuals in the total population is *N* = *N*_1_ + *N*_2_ + ⋯ + *N*_*K*_. We have *M* SNPs, whose frequencies of their coded alleles in population *k* are [ *f*_*k*1_, *f*_*k*2_, ⋯, *f*_*kM*_]. Let ***X*** be the genotype matrix of dimension *N* × *M*. The genotypic value ***X***(*n,m*) is the number of the coded allele of SNP *m* for individual *n*, where *n* = 1, 2, ⋯, *N* and *m* = 1, 2, ⋯, *M*. Typically, the number of individuals is less than the number of markers, i.e. *N* < *M*. We assume that all SNPs are under the Hardy-Weinberg equilibrium in each population. The GRM can be calculated as

1 $$ \boldsymbol{Z}=\frac{1}{M}\boldsymbol{Y}{\boldsymbol{Y}}^{\mathrm{T}}, $$

where each entry of ***Y*** is a normalized version of the coded genotype in ***X***, i.e.

2 $$ \boldsymbol{Y}\left(n,m\right)=\frac{\boldsymbol{X}\left(n,m\right)-{\mu}_m}{\sigma_m} $$

for *n* = 1, 2, ⋯, *N* and *m* = 1, 2, ⋯, *M*. Here, *μ*_*m*_ and *σ*_*m*_ denote the genotypic mean and standard deviation of SNP *m* in the total population, respectively. It can be shown that *μ*_*m*_ and *σ*_*m*_ relate to the population structure and allele frequencies as follows (Supplemental Text S1)

3 $$ {\mu}_m=2\frac{\sum_{k=1}^K{N}_k{f}_{km}}{N}, $$

4 $$ {\sigma}_m^2=2\frac{\sum_{k=1}^K{N}_k{f}_{km}\left(1-{f}_{km}\right)}{N}+4\frac{\sum_{k=1}^K{\sum}_{{}_{k\ne l}{}^{l=1}}^K{N}_k{N}_l}{N^2}{\left({f}_{km}-{f}_{lm}\right)}^2. $$

The coded-allele frequency of SNP *m* in the total population can be found as *f*_*m*_ = *μ*_*m*_/2, where *m* = 1, 2, ⋯, *M*. The GRM is of dimension *N* × *N*, whose diagonal elements are genotypic variance of individuals and off-diagonal elements are genotypic covariance between two individuals. It should be noted that genotypes follow mixed binomial distributions, and elements of ***Z*** are sample variances and covariance computed from the genotype data. The PCA analysis of population stratification is based on the eigen-analysis of the observed GRM ***Z***.

In practice, *μ*_*m*_ and *σ*_*m*_ are unknown, and therefore sample mean ${\hat{\mu}}_m$ and sample standard deviation ${\hat{\sigma}}_m$ or some other quantities similar are used instead. Usually, ${\hat{\mu}}_m=2{\hat{f}}_m$ is used for the centralization in (2). In EIGENSOFT, $\sqrt{{\hat{f}}_m\left(1-{\hat{f}}_m\right)}$ is adopted for the normalization in (2), while $\sqrt{2{\hat{f}}_m\left(1-{\hat{f}}_m\right)}$ is employed in GCTA [22]. Different estimates of the allele frequency *f*_*m*_ were suggested as well [5, 6]. In the following theoretical derivations, we assume that *μ*_*m*_ and *σ*_*m*_ are known for the sake of simplicity. This will bring about clear connections between variance and covariance elements of the EGRM and allele frequencies of the SNPs used in the analysis. The connections further provide clues and insights for understanding the effect of rare variants on the PCA of population stratification.

### Expected genetic relationship matrix

We assume that all individuals are unrelated. When the number of markers *M* goes large, the sample variance and covariance elements in the GRM will converge to their mathematical expectations in probability due to the law of large numbers. We denote the EGRM as *Z*, which is the expectation of the GRM ***Z***. Without loss of generality, we assume that individuals are ordered according to their population memberships. As such, the first *N*_1_ rows and columns correspond to individuals from population 1, the next *N*_2_ rows and columns are from population 2, and so on. Thus, the EGRM can be partitioned into a block matrix consisting of *K* × *K* submatrices

5 $$ Z=\left(\begin{array}{cccc}{Z}_{11}& {Z}_{12}& \dots & {Z}_{1K}\\ {}{Z}_{12}^{\mathrm{T}}& {Z}_{22}& \dots & {Z}_{2K}\\ {}\vdots & \vdots & \ddots & \vdots \\ {}{Z}_{1K}^{\mathrm{T}}& {Z}_{2K}^{\mathrm{T}}& \dots & {Z}_{KK}\end{array}\right). $$

Diagonal sub-matrices of the EGRM *Z* have the following structure

6 $$ {Z}_{kk}=\left(\begin{array}{cccc}{z}^k& {z}^{kk}& \dots & {z}^{kk}\\ {}{z}^{kk}& {z}^k& \dots & {z}^{kk}\\ {}\vdots & \vdots & \ddots & \vdots \\ {}{z}^{kk}& {z}^{kk}& \dots & {z}^k\end{array}\right),k=1,2,...,K. $$

Here, diagonal elements of the submatrix *Z*_*kk*_ are of the mathematical form

7 $$ {z}^k=\frac{1}{M}\sum \limits_{m=1}^M\frac{2{f}_{km}\left(1-{f}_{km}\right)+{\left(2{f}_{km}-{\mu}_m\right)}^2}{\sigma_m^2} $$

which is the genotypic variance of individuals from population *k*. All off-diagonal elements share the form

8 $$ {z}^{kk}=\frac{1}{M}\sum \limits_{m=1}^M\frac{{\left(2{f}_{km}-{\mu}_m\right)}^2}{\sigma_m^2} $$

which is the genotypic covariance between two individuals from population *k*.

The off-diagonal sub-matrices of the EGRM *Z* are structured as follows

9 $$ {Z}_{kl}=\left(\begin{array}{cccc}{z}^{kl}& {z}^{kl}& \dots & {z}^{kl}\\ {}{z}^{kl}& {z}^{kl}& \dots & {z}^{kl}\\ {}\vdots & \vdots & \ddots & \vdots \\ {}{z}^{kl}& {z}^{kl}& \dots & {z}^{kl}\end{array}\right),k\ne l. $$

Elements of *Z*_*kl*_ share the value

10 $$ {z}^{kl}=\frac{1}{M}\sum \limits_{m=1}^M\frac{\left(2{f}_{km}-{\mu}_m\right)\left(2{f}_{lm}-{\mu}_m\right)}{\sigma_m^2} $$

which is the genotypic covariance between one individual from population *k* and one from population *l*. Details of the derivations are presented in Supplemental Text S2.

The EGRM *Z*, the mathematical expectation of GRM ***Z***, depends only on the population sizes *N*_1_, *N*_2_, ⋯, *N*_*K*_ and allele frequencies of the *M* SNPs in *K* populations [ *f*_*k*1_, *f*_*k*2_, ⋯, *f*_*kM*_], *k* = 1, 2, ⋯, *K*. Here, we treat the SNP number *M* and the allele frequencies as fixed numbers. A theoretical formulation of the PCA that considers genotypic values as a random vector and allele frequencies in different populations being random was proposed in Ma and Amos, 2010 [23]. Based on different assumptions, a genotypic variance-covariance matrix of the same structure was attained; nevertheless, elements of the EGRM are different from those of the variance-covariance matrix in [23].

### Rare variants on the eigenvalues

Carrying out eigen-decomposition on the EGRM, it can be shown that there are *N*_*k*_ − 1 eigenvalues of value *z*^*k*^ − *z*^*kk*^, for *k* = 1, 2, ⋯, *K*. Here,

$$ {z}^k-{z}^{kk}=\frac{1}{M}{\sum}_{m=1}^M\frac{2{f}_{km}\left(1-{f}_{km}\right)}{\sigma_m^2}. $$

The sum of the *N* − *K* eigenvalues is

11 $$ {\sum}_{k=1}^K\left({N}_k-1\right)\left({z}^k-{z}^{kk}\right)=\frac{1}{M}{\sum}_{m=1}^M{\sum}_{k=1}^K\frac{2\left({N}_k-1\right){f}_{km}\left(1-{f}_{km}\right)}{\sigma_m^2} $$

Clearly, variations in the *N* − *K* PCs are entirely intra-population variance of the *K* populations. The sum of the other *K* eigenvalues is

12 $$ \sum \limits_{k=1}^K{\lambda}_k={\sum}_{k=1}^K{N}_k{z}^{kk}+{\sum}_{k=1}^K\left({z}^k-{z}^{kk}\right)={\sigma}_{\mathrm{B}}^2+{\sigma}_{\mathrm{W}}^2, $$

where

$$ {\sigma}_{\mathrm{B}}^2={\sum}_{k=1}^K{N}_k{z}^{kk}=\frac{1}{M}{\sum}_{m=1}^M{\sum}_{k=1}^K\frac{N_k{\left(2{f}_{km}-{\mu}_m\right)}^2}{\sigma_m^2} $$

represents the inter-population variance component and

$$ {\sigma}_{\mathrm{W}}^2={\sum}_{k=1}^K\left({z}^k-{z}^{kk}\right)=\frac{1}{M}{\sum}_{m=1}^M{\sum}_{k=1}^K\frac{2{f}_{km}\left(1-{f}_{km}\right)}{\sigma_m^2} $$

stands for the intra-population variance component. Here, the intra-population covariance *z*^*kk*^ of the EGRM factor in the *K* PCs as the inter-population variance after the eigen-decomposition. Note that all inter-population variance is solely contained in the *K* PCs. Hence, it is sufficient to conduct the population stratification analysis based on the *K* PCs alone.

Given a set of SNPs, the divergence among the *K* populations can be measured by the ratio of the two variance components, i.e.

13 $$ {\mathrm{F}}_{\mathrm{PC}}=\frac{\sigma_{\mathrm{B}}^2}{\sigma_{\mathrm{W}}^2}. $$

Its normalized version can be defined as

14 $$ {\mathrm{F}}_{\mathrm{PC}}^{\ast }=\frac{\sigma_{\mathrm{B}}^2}{\sigma_{\mathrm{B}}^2+{\sigma}_{\mathrm{W}}^2}, $$

which measures the portion of inter-population variance in the *K* population informative PCs and takes a value between 0 and 1. The larger the F_PC_ and ${\mathrm{F}}_{\mathrm{PC}}^{\ast }$ are, the more divergence among the populations.

Note that ${\mu}_m=2{f}_m=\frac{2}{N}{\sum}_{k=1}^K{N}_k{f}_{km}$, terms in ${\sigma}_{\mathrm{B}}^2$ are quadratic functions of *f*_*km*_, *k* = 1, 2, ⋯, *K*, *m* = 1, 2, ⋯, *M*. However, terms in ${\sigma}_{\mathrm{W}}^2$ are linear and quadratic functions of the frequencies. Therefore, F_PC_ will decrease when frequencies of the coded alleles become smaller, see Supplemental Text S3 for more details. As a result, instead of improving the population stratification analysis, using rare variants will deteriorate the analysis performance. Meanwhile, since ${\sigma}_{\mathrm{B}}^2$ decreases faster than ${\sigma}_{\mathrm{W}}^2$, the *K* eigenvalues will be closer to the other *N* − *K* eigenvalues when frequencies of the coded alleles become smaller. Thus, the percent of variance explained by the *K* PCs will be smaller.

It can be shown that among the *K* eigenvalues, *K* − 1 are of large values and one small. When intra-population variance *z*^*k*^ − *z*^*kk*^ of the *K* populations are equal, all inter-population variance is contained in the largest *K* − 1 eigenvalues. In addition, when the sample size is large and the portions of populations remain, inter-population variance contained in the small eigenvalue is negligible, almost all information on the population structure is contained in the largest *K* − 1 PCs.

For cases with two populations, it can be shown that the two eigenvalues are

$$ {\lambda}_1=\frac{N_1{z}^{11}}{2}+\frac{N_2{z}^{22}}{2}+\frac{z^1-{z}^{11}}{2}+\frac{z^2-{z}^{22}}{2}+\frac{\sqrt{a}}{2}, $$

$$ {\lambda}_2=\frac{N_1{z}^{11}}{2}+\frac{N_2{z}^{22}}{2}+\frac{z^1-{z}^{11}}{2}+\frac{z^2-{z}^{22}}{2}-\frac{\sqrt{a}}{2}, $$

where

$$ a={\left[\left({z}^1-{z}^{11}\right)-\left({z}^2-{z}^{22}\right)+{N}_1{z}^{11}-{N}_2{z}^{22}\right]}^2+4{N}_1{N}_2{\left({z}^{12}\right)}^2. $$

When inter-population variance of the two populations are equal, i.e. ${z}^1-{z}^{11}={z}^2-{z}^{22}={\sigma}_{\mathrm{W}}^2/2$, we have

$$ {\lambda}_1={N}_1{z}^{11}+{N}_2{z}^{22}+\frac{\sigma_{\mathrm{W}}^2}{2}, $$

$$ {\lambda}_2=\frac{\sigma_{\mathrm{W}}^2}{2}. $$

That is, all information on the population structure is contained in the largest PC. All proofs are presented in Supplemental Text S4.

### Rare variants on the population distance

Suppose that ***x***_*k*_, *k* = 1, 2, ⋯, *K* are the eigenvectors associated with the *K* eigenvalues containing inter-population variance. We can represent each individual as a point in the *K*-dimension space. Vector $\sqrt{\lambda_k}{\boldsymbol{x}}_k$ consists of coordinates of *N* individuals in the *k*-th dimension. Average value $\sqrt{\lambda_k}{\boldsymbol{x}}_k^{\mathrm{T}}{\mathbf{1}}_N/N$ represents center of the total population in the *k*-th dimension, where **1**_*N*_ is a column vector of dimension *N* and with each element as 1. Due to the structure of *Z*, individuals from the same population share the same coordinates in the *K*-dimension space, and the common points denote the representative points of the populations, or centers of the populations [23]. We define

$$ {d}^2={\sum}_{k=1}^K{\left[\sqrt{\lambda_k}{\boldsymbol{x}}_k-\left(\sqrt{\lambda_k}{\boldsymbol{x}}_k^{\mathrm{T}}{\mathbf{1}}_N/N\right){\mathbf{1}}_N\right]}^{\mathrm{T}}\left[\sqrt{\lambda_k}{\boldsymbol{x}}_k-\left(\sqrt{\lambda_k}{\boldsymbol{x}}_k^{\mathrm{T}}{\mathbf{1}}_N/N\right){\mathbf{1}}_N\right] $$

$$ ={\sum}_{k=1}^K{\lambda}_k-\frac{1}{N}{\mathbf{1}}_N^{\mathrm{T}}Z{\mathbf{1}}_N $$

15 $$ ={\sum}_{k=1}^K{\lambda}_k-\frac{1}{N}{\sum}_{k=1}^K{N}_k\left({z}^k-{z}^{kk}\right) $$

which measures the population divergence as the sum of squared distances among populations. The proof is shown in Supplemental Text S5. Here, the second term in (15) is an average intra-population variance. As explained earlier that when allele frequencies become smaller, the *K* eigenvalues decrease. Hence, the population distance *d*^2^ is smaller when using rare SNPs compared to common ones.

### The 1000 genomes project data

We used genotype data from the 1000 Genomes Project to validate our theoretical results. Genotype data used in this work was obtained from Phase 3 version 5a of the 1000 Genomes Project [16, 17], which contains 84.4 million genetic markers and 2504 individuals from EUR, EAS, SAS, AFR and AMR. We extracted biallelic SNPs that are polymorphic in the total population. In summary, there are 77,279,863 SNPs; 5,261,820 are common (0.1 < MAF ≤ 0.5), 6,770,457 are low-frequency (0.01 < MAF ≤ 0.1), and 65,247,586 are rare (0.0001 < MAF ≤ 0.01). Genotype data were converted to PLINK format with VCFtools [24]. The SNPs were divided into six frequency bins according to their MAFs, as shown in Table 1. For each bin, we randomly sampled approximately 100,000 SNPs using PLINK for the population stratification analyses. Here, MAFs of the SNPs in the total population were used, hence their frequencies in the five populations may be different and may not be in the same bins as in the total population. For SNPs in bin 5 and 6, they are polymorphic in the total population and may not be polymorphic in all of the five populations. PCAs were carried out, with GRMs computed by EIGENSOFT and PCAs on EGRMs conducted using GCTA. Default parameters were used when analyzing with EIGENSOFT, which excluded 68 and 116 outliers in the analyses of the data from frequency bin 5 and 6, respectively.

**Table 1:** Summary of SNPs from the 1000 Genomes Project data

	Common SNP				Low-frequency SNP	Rare SNP
MAF	(0.4,0.5]	(0.3,0.4]	(0.2,0.3]	(0.1,0.2]	(0.01,0.1]	(0. 0001,0.01]
Pop (N)	Bin 1	Bin 2	Bin 3	Bin 4	Bin 5	Bin 6
EUR (503)	995,352	1,048,669	1,190,239	1,581,788	3,717,490	13,531,139
EAS (504)	970,359	1,010,549	1,130,961	1,440,178	2,982,582	14,189,976
AMR (347)	1,004,970	1,068,395	1,234,095	1,613,443	4,827,083	16,092,172
SAS (489)	1,001,330	1,077,620	1,239,727	1,626,183	3,989,855	15,562,799
AFR (661)	981,929	1,097,944	1,436,771	2,403,901	8,852,607	24,044,176
Total (2504)	1,023,570	1,105,365	1,308,728	1,824,157	6,770,457	65,247,586

*MAF* minor allele frequency, *Pop* population, *EUR* European, *EAS* East Asian, *AMR* American, *SAS* South Asian, *AFR* African

## Results

### Theoretical and empirical EGRMs

To calculate the theoretical results (5)–(10), we computed MAFs of the SNPs with PLINK. Values of variance *z*^*k*^ and covariance *z*^*kk*^, *z*^*kl*^, *k*, *l* = 1, 2, ⋯*K*, were calculated as in (7), (8), and (10), respectively, in which *μ*_*m*_ was computed with (3) and ${\sigma}_m^2=2{f}_m\left(1-{f}_m\right)$, *m* = 1, 2, ⋯*M*. Values of *z*^*k*^ and *z*^*kk*^ for the five populations with SNPs from the six frequency bins are presented in Table 2. Absolute values of the inter-population covariance *z*^*kl*^ are much smaller and the results are shown in Supplemental Tables S1–6.

**Table 2:** Theoretical and empirical values of the variance and covariance elements of EGRMs

	MAF	EUR	EAS	AMR	SAS	AFR
***z***^***k***^	(0.4,0.5]	1.06/0.97	1.11/1.02	1.04/0.97	1.04/0.96	1.15/1.05
	(0.3,0.4]	1.06/0.97	1.11/1.02	1.04/0.97	1.04/0.96	1.14/1.05
	(0.2,0.3]	1.05/0.96	1.09/1.00	1.03/0.97	1.03/0.96	1.17/1.07
	(0.1,0.2]	0.99/0.91	1.01/0.93	0.98/0.93	0.99/0.92	1.32/1.22
	(0.01,0.1]	0.61/0.57	0.50/0.47	0.73/0.70	0.56/0.53	2.50/2.37
	(0.0001,0.01]	0.71/0.71	0.94/0.94	0.82/0.82	0.98/0.98	1.46/1.46
***z***^***kk***^	(0.4,0.5]	0.13/0.11	0.22/0.20	0.08/0.07	0.08/0.07	0.30/0.28
	(0.3,0.4]	0.13/0.11	0.22/0.20	0.07/0.06	0.08/0.07	0.29/0.26
	(0.2,0.3]	0.12/0.11	0.21/0.19	0.07/0.06	0.08/0.07	0.27/0.24
	(0.1,0.2]	0.11/0.10	0.18/0.17	0.07/0.06	0.08/0.07	0.27/0.25
	(0.01,0.1]	0.06/0.05	0.08/0.07	0.04/0.03	0.05/0.04	0.25/0.23
	(0.0001,0.01]	0.004/0.002	0.005/0.003	0.004/0.002	0.005/0.003	0.011/0.008

The first values are theoretical values of the variance and covariance, and second values are empirical values

To obtain the empirical values of variance *z*^*k*^, as well as covariance *z*^*kk*^ and *z*^*kl*^, we first computed GRMs with SNPs from the six bins using EIGENSOFT. Each GRM included *N*(*N* + 1)/2 variance and covariance terms of *N* individuals based on the observed genotype data. Empirical value of *z*^*k*^ was computed as the average variance of the *N*_*k*_ individuals from population *k*. The empirical value of *z*^*kk*^ is the average covariance of *N*_*k*_(*N*_*k*_ − 1)/2 pairs of individuals from population *k*. Lastly, the value of *z*^*kl*^ is the average covariance of *N*_*k*_*N*_*l*_ pairs of individuals, one from population *k* and one from population *l*. The results of *z*^*k*^ and *z*^*kk*^ are shown in Table 2, and those of *z*^*kl*^ are presented in Supplemental Tables S1–6.

We can see that across the six frequency bins, theoretical values of *z*^*k*^, *z*^*kk*^, and *z*^*kl*^ predicted by (7), (8), and (10), respectively, are close to their empirical values. When MAFs of the SNPs become smaller, intra-population covariance *z*^*kk*^ decreases. For example, *z*^*kk*^ was 0.2 for EAS with SNPs whose MAFs are between 0.4 and 0.5, which reduced to 0.003 in the sixth bin that included rare SNPs only. A similar pattern can be observed for the other four populations. F_PC_ was estimated by (13) for the six bins, where empirical values of *z*^*k*^ and *z*^*kk*^ were used. The F_PC_ decreases from 93.85 in bin 1 to 55.01 in bin 5, and further to 1.83 in bin 6. Thus the divergence among the populations is much larger when measured by common SNPs than by rare ones.

### PCAs of the 1000 genomes project data

**Fig. 1:**
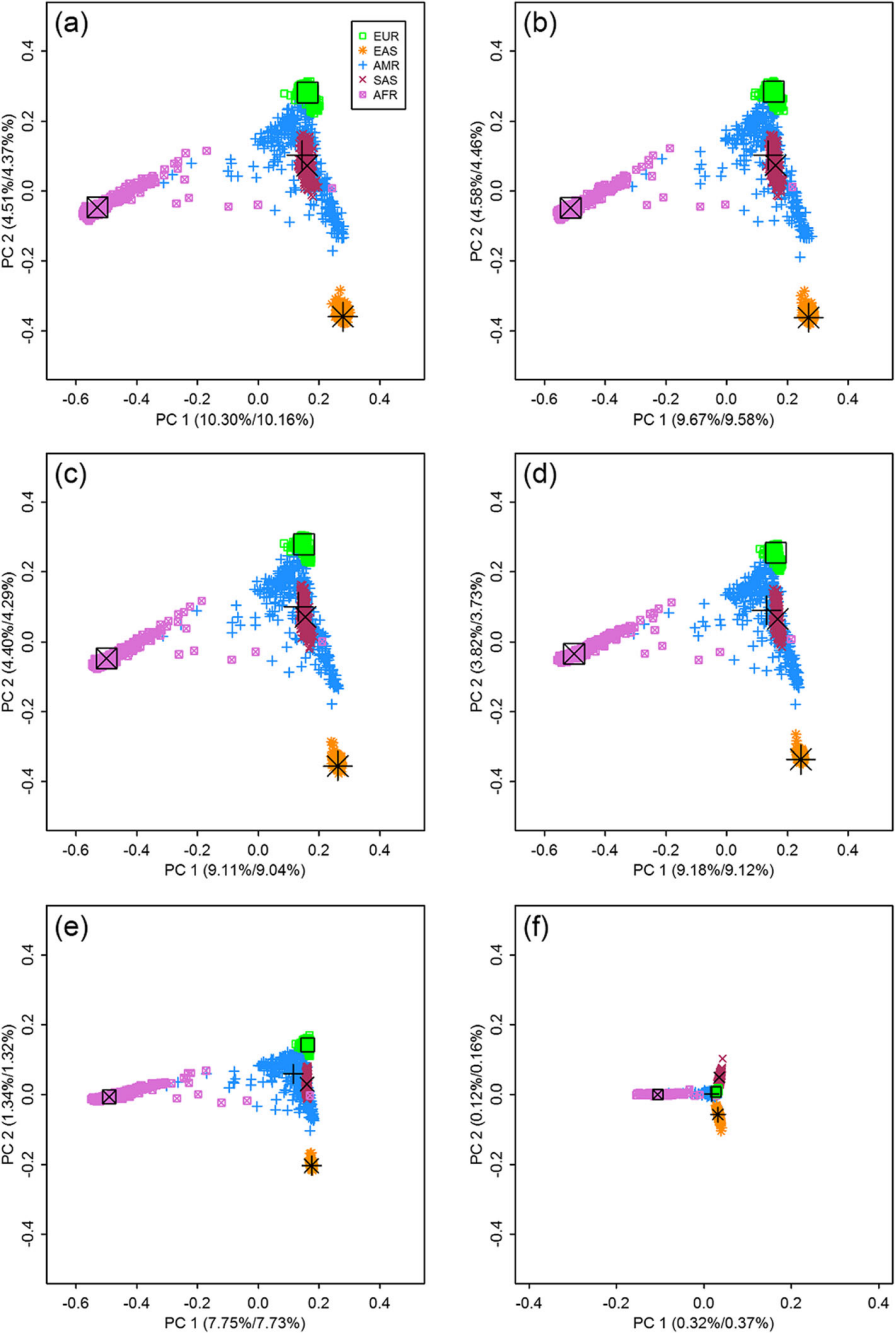
Scatter plots and representative points with SNPs from six MAF bins, PC 1 vs. PC 2. (**a**) 0.4 < MAF ≤ 0.5 (**b**) 0.3 < MAF ≤ 0.4 (**c**) 0.2 < MAF ≤ 0.3 (**d**) 0.1 < MAF ≤ 0.2 (**e**) 0.01 < MAF ≤ 0.1 (**f**) 0.0001 < MAF ≤ 0.01. EUR: European, EAS: East Asian, AMR: American, SAS: South Asian, AFR: African. The first values in brackets are the percentages of variance explained from the PCAs of GRMs; and the second values are from the PCAs of EGRMs. Large symbols in black are the representative points of the five populations

**Fig. 2:**
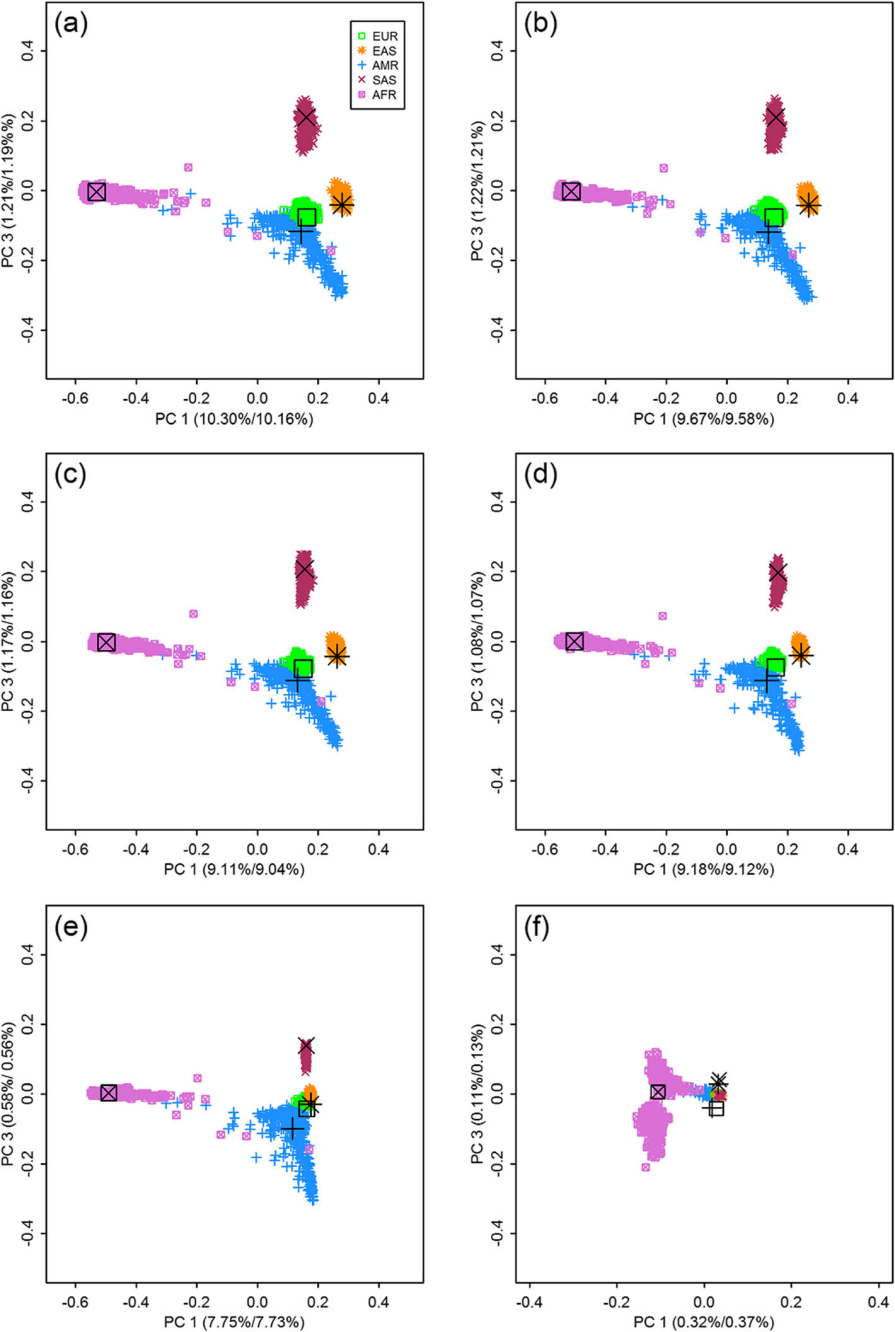
Scatter plots and representative points with SNPs from six MAF bins, PC 1 vs. PC 3. (**a**) 0.4 < MAF ≤ 0.5 (**b**) 0.3 < MAF ≤ 0.4 (**c**) 0.2 < MAF ≤ 0.3 (**d**) 0.1 < MAF ≤ 0.2 (**e**) 0.01 < MAF ≤ 0.1 (**f**) 0.0001 < MAF ≤ 0.01. EUR: European, EAS: East Asian, AMR: American, SAS: South Asian, AFR: African. The first values in brackets are the percentages of variance explained from the PCAs of GRMs; and the second values are from the PCAs of EGRMs. Large symbols in black are the representative points of the five populations

With genotypes of SNPs from each frequency bin, we carried out PCAs of population stratification by EIGENSOFT, which was essentially based on the eigen-analysis of the observed GRMs. Scatter plots of the largest three PCs are shown in Figs. 1 and 2, where eigenvectors were scaled by square roots of their corresponding eigenvalues.

From Figs. 1 and 2, we can see patterns of population structure computed with common and less-frequency SNPs. For example, Figs. 1a-e and 2a-e displayed similar patterns, whereas the scatter plots based on rare SNPs differed significantly. For example, AMR and SAS are separated mostly by the third PC with common SNPs, while they are distinguished by the second PC with rare ones. The third PC from rare SNPs reveals mostly substructure of AFR, likely because more rare SNPs are polymorphic in AFR than in other populations. Portions of variance explained by the largest five PCs decrease from 17.09% in bin 1 to 10.41% in bin 5, and it falls dramatically to 0.74% with rare SNPs only. As a result, the five populations are more closely distributed around the origin in Figs. 1f and 2f, compared with those in Figs. 1a-e and 2a-e. Clearly, common variants show much better performance in dissecting the population structure than rare variants do.

### PCAs of EGRMs

For each frequency bin, we also constructed a EGRM with structure as described in (5), (6), and (9), whose variance and covariance elements were their theoretical values calculated by (7), (8), and (10), respectively. We conducted PCAs of the EGRMs using GCTA, and scatter plots of the largest three PCs shown in Figs. 1 and 2. Large symbols in black are representative points or centers of the five continental populations from eigen-analyses of the EGRMs. Similarly, coordinates were scaled by square roots of their eigenvalues.

Upon comparing the representative points in Figs. 1 and 2, we can see that distances between populations decrease as the SNPs change from common to rare. Sum of the squared distance *d*^2^ was calculated for the six frequency bins by (15), where *λ*_*k*_, *k* = 1, 2, ⋯*K* were the eigenvalues of the EGRM *Z* and *z*^*k*^, *z*^*kk*^, *k* = 1, 2, ⋯*K* were their theoretical values. The *d*^2^ decreases from 444.38 in bin 1 to 254.10 in bin 5, and further to 17.83 in bin 6.

In addition, when portions of variance explained by the PCs become small, deviations between the representative points of the populations and true centers of the populations can be observed. This is particularly evident in the scatter plots with rare SNPs. In the PCAs of a single population, such deviations are more obvious when percents of variance explained by the largest PCs are much smaller.

## Discussion

We showed that all information about the population structure is contained in *K* PCs. Genotypic variance explained by the *K* PCs can be further decomposed into the intra-population variance ${\sigma}_{\mathrm{W}}^2$ and inter-population variance ${\sigma}_{\mathrm{B}}^2$. Using more SNPs will improve convergence of the realized GRM to its mathematical expectation, i.e. the EGRM. As a result, individuals belonging to the same population will be more closely distributed around the representative point or center of the population on the PC-PC plots. On the other hand, note that ${\sigma}_{\mathrm{B}}^2$ is the average inter-population variance contributed from *M* SNPs. When rare variants are used, adding more SNPs will not increase the average level of ${\sigma}_{\mathrm{B}}^2$, hence neither the ratio F_PC_ nor the sum of squared distances *d*^2^ will improve. For same reason, using a combination of common and rare SNPs will result in lower F_PC_ and *d*^2^ compared with using common SNPs only and therefore result in worse performance.

In the case where there is one SNP, our F_PC_ and ${\mathrm{F}}_{\mathrm{PC}}^{\ast }$ can be further reduced to

$$ {\mathrm{F}}_{\mathrm{PC}}=\frac{{\mathrm{F}}_{\mathrm{PC}}^{\ast }}{1-{\mathrm{F}}_{\mathrm{PC}}^{\ast }}=2\frac{\sum_{k=1}^K{N}_k{\left({f}_k-f\right)}^2}{\sum_{k=1}^K{f}_k\left(1-{f}_k\right)}, $$

where *f*_*k*_ is the allele frequency in the population *k*, and *f* the frequency in the total population. The classical Wright’s fixation index F_ST_ is widely used to gauge population stratification [25], which measures the deviation from Hardy-Weinberg equilibrium at the total population level. In this case, it can be shown that

$$ \frac{{\mathrm{F}}_{\mathrm{ST}}}{1-{\mathrm{F}}_{\mathrm{ST}}}=\frac{\sum_{k=1}^K{N}_k{\left({f}_k-f\right)}^2}{\sum_{k=1}^K{N}_k{f}_k\left(1-{f}_k\right)}. $$

Therefore, our ${\mathrm{F}}_{\mathrm{PC}}^{\ast }$ is much larger than F_ST._ It is worth pointing out that we assign numeric values to genotypes as numbers of the coded alleles, hence our results are dependent on such coding scheme. F_ST_, however, does not involve in the numeric coding of genotypes. Note also that ${\mathrm{F}}_{\mathrm{PC}}^{\ast }$ measures the portion of inter-population variance in the *K* population informative PCs. After the eigen-decomposition, most of the intra-population variance associated with the other *N-K* PCs was excluded. If our F_PC_ were defined as the ratio of the inter-population variance to the intra-population variance in the *N* PCs, it would be related to F_ST_ as F_PC_ = 2F_ST_/(1 − F_ST_).

In GWAS, it is a common practice to conduct population stratification analyses using a large number of random markers [26], which usually yields satisfactory results. As shown in this work, the capability of dissecting population structure depends on the allele frequencies of markers used in the analyses, and common variants perform much better than rare ones. This is not much of a concern for GWAS because SNP panels implemented in the genotyping platforms are mostly of common ones. In sequencing studies, however, the majority of the called variants are rare, and selecting SNPs randomly will yield a large portion of rare SNPs, which will deteriorate the analysis performance. Therefore, it is necessary to restrict the selection to only the common SNPs when analyzing population stratification with sequencing data. This would also be true for controlling population stratification based on the linear mixed models [11–14].

In this work, we assumed that *μ*_*m*_ and *σ*_*m*_ are known constants in (2) in order to simplify the theoretical derivations. Our results are approximations of those when estimates of the two quantities are used. When sample size *N* is large, variations associated with ${\hat{\mu}}_m$ and ${\hat{\sigma}}_m$ are much smaller than those with the genotype data. Therefore, the mathematical expectations are largely taken with respect to the genotypes and difference between the two sets of results would be small. As shown in Table 2, the predicted values of the EGRM are close to their empirical values in the 1000 Genome Project data. We carried out additional simulation studies to evaluate the effect of lacking knowledge on *μ*_*m*_ and *σ*_*m*_. We randomly chose one SNP from each of the six frequency bins (Supplemental Table S7). Based on their MAFs observed in the five populations of the 1000 Genomes Project, we simulated genotypes of five populations each with 500 individuals. Values of *μ*_*m*_ and *σ*_*m*_ were computed with the assumption of known population structure and MAF information, and theoretical values of *z*^*k*^ and *z*^*kk*^ were then calculated. For comparison, we first estimated ${\hat{\mu}}_m$ and ${\hat{\sigma}}_m$ from the simulated genotype data. ***Y***(*n*, *m*) were normalized with ${\hat{\mu}}_m$ and ${\hat{\sigma}}_m$, and *z*^*k*^ and *z*^*kk*^ were obtained as averages of sample variance and covariance from 1000 replicates. The two sets of results are presented in Supplemental Table S8 and the differences between the two sets of results are negligible except for small differences in the results with the rare SNP.

Inferring population structure based on a large number of genome-wide markers are likely to include markers in linkage disequilibrium (LD). Practical concerns on the LD and choice of markers were extensively discussed in [5]. It is worth noting that each marker contributes to the elements in GRM additively, see eqs. S1–3 in Supplemental Text S2. Because of the linearity of expectation, our EGRM formulae as well as the eigen-analysis on the EGRM still hold when LD exists among markers. When the number of markers goes large, convergence of the GRM to EGRM will be slower with LD among markers, compared with the case that independent markers are used. Since there are always limited number of markers in the PCA practice, our EGRM and the eigen-analysis on it represent asymptotic results of the real PCA analysis.

Despite the fact that the vast majority of rare variants are population-specific, we showed that performance of the PCA of population stratification is better when based on common SNPs rather than rare ones. On the other hand, the PCA results with rare SNPs do reveal a population structure that differs from that of common SNPs. Existing methods may not exploit ancestry information embedded in the rare variants efficiently, and different approaches from those applied to common variants should be developed [26].

## Conclusions

To quantify population divergence as a function of allele frequencies of genetic markers used in the PCA analysis, we derived the expected genetic relationship matrix. We proposed F_PC_, ratio of the inter-population variance to the intra-population variance, and population distance *d*^2^ as measures of population divergence. Our theoretical results as well as the analyses of the 1000 Genomes Project data showed that employing rare variants yielded smaller F_PC_ in the *K* population informative PCs, smaller *d*^2^, and smaller portion of variance explained by the *K* PCs than those using common variants. Therefore, the PCA of population stratification performs worse with rare variants than with common ones. When analyzing population stratification with sequencing data, it is necessary to restrict the selection to only the common variants.

## Supplementary information

**Additional file 1 Text S1**. Proof of eq. (4). **Text S2**. Proofs of eqs. (5–10). **Text S3**. F_PC_ as a function of allele frequencies. **Text S4**. Proofs of rare variants on the eigenvalues. **Text S5**. Proof of eq. (15). **Table S1**. Theoretical and empirical values of the inter-population covariance of EGRM (0.4 < MAF ≤ 0.5). **Table S2**. Theoretical and empirical values of the inter-population covariance of EGRM (0.3 < MAF ≤ 0.4). **Table S3**. Theoretical and empirical values of the inter-population covariance of EGRM (0.2 < MAF ≤ 0.3). **Table S4**. Theoretical and empirical values of the inter-population covariance of EGRM (0.1 < MAF ≤ 0.2). **Table S5**. Theoretical and empirical values of the inter-population covariance of EGRM (0.01 < MAF ≤ 0.1). **Table S6**. Theoretical and empirical values of the inter-population covariance of EGRM (0.0001 < MAF ≤ 0.01). **Table S7**. MAFs of the six SNPs used in the simulations. **Table S8**. Expected variance and covariance with and without the knowledge of *μ*_*m*_ and *σ*_*m*_.

## Data Availability

The datasets analyzed during the current study are available at https://www.internationalgenome.org. The accession number at https://www.ebi.ac.uk/ena is PRJNA262923.

## Abbreviations

GWAS: Genome-wide association study
LD: Linkage disequilibrium
MAF: Minor allele frequency
PC: Principal component
PCA: Principal component analysis
SNP: Single nucleotide polymorphism
