# Supplementary material for: On rare variants in principal component analysis of population stratification
Source: BMC Genet. 2020 Mar 17;21:34. doi: 10.1186/s12863-020-0833-x (PMC7077175; doi:10.1186/s12863-020-0833-x)
Supplement: Supplementary file 1 — Additional file 1 Text S1. Proof of eq. (4). Text S2. Proofs of eqs. (5–10). Text S3. FPC as a function of allele frequencies. Text S4. Proofs of rare variants on the eigenvalues. Text S5. Proof of eq. (15). Table S1. Theoretical and empirical values of the inter-population covariance of EGRM (0.4 < MAF ≤ 0.5). Table S2. Theoretical and empirical values of the inter-population covariance of EGRM (0.3 < MAF ≤ 0.4). Table S3. Theoretical and empirical values of the inter-population covariance of EGRM (0.2 < MAF ≤ 0.3). Table S4. Theoretical and empirical values of the inter-population covariance of EGRM (0.1 < MAF ≤ 0.2). Table S5. Theoretical and empirical values of the inter-population covariance of EGRM (0.01 < MAF ≤ 0.1). Table S6. Theoretical and empirical values of the inter-population covariance of EGRM (0.0001 < MAF ≤ 0.01). Table S7. MAFs of the six SNPs used in the simulations. Table S8. Expected variance and covariance with and without the knowledge of μm and σm. [file 12863_2020_833_MOESM1_ESM.docx]

**Text S1: Proof of equation (4)**

The genotypic variance of SNP *m* can be found as

$\begin{matrix} \sigma_{m}^{2}=\sum_{k=1}^{K} \frac{N_{k}}{N}2f_{km}\left(1-f_{km}\right)+\sum_{k=1}^{K} \frac{N_{k}}{N}\left(\mu_{km}-\mu_{m}\right)^{2} \\ =\frac{N_{1}\sigma_{1m}^{2}+N_{2}\sigma_{2m}^{2}+\ldots+N_{K}\sigma_{Km}^{2}}{N}+\frac{N_{1}\mu_{1m}^{2}+N_{2}\mu_{2m}^{2}+\ldots+N_{K}\mu_{Km}^{2}}{N}-\mu_{m}^{2} \\ =\frac{N_{1}\sigma_{1m}^{2}+N_{2}\sigma_{2m}^{2}+\ldots+N_{K}\sigma_{Km}^{2}}{N} \\ +\frac{NN_{1}\mu_{1m}^{2}+NN_{2}\mu_{2m}^{2}+\ldots+NN_{K}\mu_{Km}^{2}-N_{1}^{2}\mu_{1m}^{2}-N_{2}^{2}\mu_{2m}^{2}-\ldots-N_{K}^{2}\mu_{Km}^{2}-2N_{1}N_{2}\mu_{1m}\mu_{2m}}{N^{2}} \\ +\frac{-\ldots-2N_{1}N_{K}\mu_{1m}\mu_{Km}-2N_{2}N_{3}\mu_{2m}\mu_{3m}-\ldots-2N_{2}N_{K}\mu_{2m}\mu_{Km}-\ldots-2N_{K-1}N_{K}\mu_{\left(K-1\right)m}\mu_{Km}}{N^{2}} \\ =\frac{N_{1}\sigma_{1m}^{2}+N_{2}\sigma_{2m}^{2}+\ldots+N_{K}\sigma_{Km}^{2}}{N} \\ +\frac{\left(N_{1}+N_{2}+\ldots+N_{K}\right)N_{1}\mu_{1m}^{2}+\left(N_{1}+N_{2}+\ldots+N_{K}\right)N_{2}\mu_{2m}^{2}+\ldots+\left(N_{1}+N_{2}+\ldots+N_{K}\right)N_{K}\mu_{Km}^{2}}{N^{2}} \\ +\frac{-N_{1}^{2}\mu_{1m}^{2}-N_{2}^{2}\mu_{2m}^{2}-\ldots-N_{K}^{2}\mu_{Km}^{2}-2N_{1}N_{2}\mu_{1m}\mu_{2m}}{N^{2}} \\ +\frac{-\ldots-2N_{1}N_{K}\mu_{1m}\mu_{Km}-2N_{2}N_{3}\mu_{2m}\mu_{3m}-\ldots-2N_{2}N_{K}\mu_{2m}\mu_{Km}-\ldots-2N_{K-1}N_{K}\mu_{\left(K-1\right)m}\mu_{Km}}{N^{2}} \\ =\frac{\sum_{k=1}^{K} N_{k}\sigma_{km}^{2}}{N}+\frac{\sum_{k=1}^{K} \sum_{\begin{matrix} l=1 \\ l\neq k \end{matrix}}^{K} N_{k}N_{l}}{N^{2}}\left(\mu_{km}-\mu_{lm}\right)^{2} \end{matrix}$ where $\sigma_{km}^{2}=2f_{km}\left(1-f_{km}\right)$ and $\mu_{km}=2f_{km},k=1,2,...,K$.

**Text S2: Proofs of equations (5-10)**

The GRM can be partitioned similarly as follows

$$\boldsymbol{Z}=\left( \begin{matrix} \boldsymbol{Z}_{11} & \boldsymbol{Z}_{12} & \cdots& \boldsymbol{Z}_{1K} \\ \boldsymbol{Z}_{12}^{T} & \boldsymbol{Z}_{22} & \cdots& \boldsymbol{Z}_{2K} \\ \cdots& \cdots& \ddots& \cdots\\ \boldsymbol{Z}_{1K}^{T} & \boldsymbol{Z}_{2K}^{T} & \cdots& \boldsymbol{Z}_{KK} \end{matrix} \right)$$

Diagonal elements of sub-matrix $\text{Z}_{kk}$

$\text{Z}_{kk}(i',i')=\frac{1}{M}\sum_{m=1}^{M} \frac{\left(\text{X}(i,m)-\mu_{m}\right)^{2}}{\sigma_{m}^{2}},i\in S_{k}$ (S1)

is the sample variance of individual *i* in population *k*, where $i'$ is the index of individual *i* in ***Z****_kk_* and $S_{k}$ is the set that includes indices of individuals in population *k.*

Off-diagonal elements of the sub-matrix $\text{Z}_{kk}$

$\text{Z}_{kk}(i',j')=\frac{1}{M}\sum_{m=1}^{M} \frac{\left(\text{X}(i,m)-\mu_{m}\right)(\text{X}(j,m)-\mu_{m})}{\sigma_{m}^{2}},i,j\in S_{k},i\neq j$ (S2)

is the sample covariance between individuals *i* and *j* in population *k*, where $i'$ and $j'$ are the indices of individuals *i* and *j* in ***Z****_kk_*, respectively*.*

Elements of the sub-matrix $\text{Z}_{kl}$

$\text{Z}_{kl}(i',j')=\frac{1}{M}\sum_{m=1}^{M} \frac{\left(\text{X}(i,m)-\mu_{m}\right)(\text{X}(j,m)-\mu_{m})}{\sigma_{m}^{2}},i\in S_{k},j\in S_{l},k\neq l$ (S3)

is the sample covariance between individual *i* in population *k* and individual *j* in population *l*, where $i'$ and $j'$ are the indices of individuals *i* and *j* in ***Z****_kl_*, respectively.

The expected genotypic variance of an individual in population *k*

$$\begin{matrix} z^{k}=\frac{1}{M}\text{E}\left\{\sum_{m=1}^{M} \frac{\left(\text{X}(i,m)-\mu_{m}\right)^{2}}{\sigma_{m}^{2}}\right\}=\frac{1}{M}\sum_{m=1}^{M} \frac{\text{E}[\left(\text{X}(i,m)-\mu_{m}\right)^{2}]}{\sigma_{m}^{2}} \\ =\frac{1}{M}\sum_{m=1}^{M} \frac{\text{E}\left[\left((\text{X}(i,m)-\mu_{km})+\left(\mu_{km}-\mu_{m}\right)\right)^{2}\right]}{\sigma_{m}^{2}} \\ =\frac{1}{M}\sum_{m=1}^{M} \frac{\text{E}\left[(\text{X}(i,m)-\mu_{km})^{2}\right]+\left(\mu_{km}-\mu_{m}\right)^{2}}{\sigma_{m}^{2}} \\ =\frac{1}{M}\sum_{m=1}^{M} \frac{\sigma_{km}^{2}+\left(\mu_{km}-\mu_{m}\right)^{2}}{\sigma_{m}^{2}} \\ =\frac{1}{M}\sum_{m=1}^{M} \frac{2f_{km}\left(1-f_{km}\right)+\left(2f_{km}-\mu_{m}\right)^{2}}{\sigma_{m}^{2}} \end{matrix}$$

The expected covariance between individuals *i* and *j* in population *k*

$$\begin{matrix} z^{kk}=\frac{1}{M}\text{E}\left\{\sum_{m=1}^{M} \frac{(\text{X}(i,m)-\mu_{m})(\text{X}(j,m)-\mu_{m})}{\sigma_{m}^{2}}\right\} \\ =\frac{1}{M}\sum_{m=1}^{M} \frac{\text{E}\left\{\left[(\text{X}(i,m)-\mu_{km})+(\mu_{km}-\mu_{m})\right]\left[(\text{X}(j,m)-\mu_{km})+(\mu_{km}-\mu_{m})\right]\right\}}{\sigma_{m}^{2}} \\ =\frac{1}{M}\sum_{m=1}^{M} \frac{\left(\mu_{km}-\mu_{m}\right)^{2}}{\sigma_{m}^{2}} \\ =\frac{1}{M}\sum_{m=1}^{M} \frac{\left(2f_{km}-\mu_{m}\right)^{2}}{\sigma_{m}^{2}} \end{matrix}$$

The expected covariance between individual *i* in population *k* and individual *j* in population *l*

$$\begin{matrix} z^{kl}=\frac{1}{M}\text{E}\left\{\sum_{m=1}^{M} \frac{(\text{X}(i,m)-\mu_{m})(\text{X}(j,m)-\mu_{m})}{\sigma_{m}^{2}}\right\} \\ =\frac{1}{M}\sum_{m=1}^{M} \frac{\text{E}\left\{\left[(\text{X}(i,m)-\mu_{km})+(\mu_{km}-\mu_{m})\right]\left[(\text{X}(j,m)-\mu_{lm})+(\mu_{lm}-\mu_{m})\right]\right\}}{\sigma_{m}^{2}} \\ =\frac{1}{M}\sum_{m=1}^{M} \frac{\left(\mu_{km}-\mu_{m}\right)\left(\mu_{lm}-\mu_{m}\right)}{\sigma_{m}^{2}} \\ =\frac{1}{M}\sum_{m=1}^{M} \frac{\left(2f_{km}-\mu_{m}\right)\left(2f_{lm}-\mu_{m}\right)}{\sigma_{m}^{2}} \end{matrix}$$

**Text S3: F_PC_ as a function of allele frequencies**

The inter-population variance $\sigma_{B}^{2}$ depends on the allele frequencies as follows

$$\sigma_{B}^{2}=\frac{1}{M}\sum_{m=1}^{M} \sum_{k=1}^{K} \frac{N_{k}\left( 2f_{km}-\mu_{m} \right)^{2}}{\sigma_{m}^{2}}$$

$$=\frac{1}{M}\sum_{m=1}^{M} \frac{1}{\sigma_{m}^{2}}\sum_{k=1}^{K} N_{k}\left( 4f_{km}^{2}-4f_{km}\mu_{m}+\mu_{m}^{2} \right)$$

$$=\frac{1}{M}\sum_{m=1}^{M} \frac{1}{\sigma_{m}^{2}}\left( \sum_{k=1}^{K} 4N_{k}f_{km}^{2}-2\mu_{m}\sum_{k=1}^{K} 2N_{k}f_{km}+\mu_{m}^{2}\sum_{k=1}^{K} N_{k} \right)$$

$$=\frac{1}{M}\sum_{m=1}^{M} \frac{1}{\sigma_{m}^{2}}\left( \sum_{k=1}^{K} 4N_{k}f_{km}^{2}-{N\mu}_{m}^{2} \right)$$

where the last equality follows from the identity

$$\sum_{k=1}^{K} 2N_{k}f_{km}=2Nf_{m}=N\mu_{m}$$

The intra-population variance $\sigma_{W}^{2}$ depends on the allele frequencies as follows

$$\sigma_{W}^{2}=\frac{1}{M}\sum_{m=1}^{M} \frac{1}{\sigma_{m}^{2}}\sum_{k=1}^{K} 2f_{km}\left( 1-f_{km} \right)$$

$$=\frac{1}{M}\sum_{m=1}^{M} \frac{1}{\sigma_{m}^{2}}\sum_{k=1}^{K} \left( 2f_{km}-2f_{km}^{2} \right)$$

Therefore,

$$F_{\mathrm{PC}}=\frac{\frac{1}{M}\sum_{m=1}^{M} \frac{1}{\sigma_{m}^{2}}\left( \sum_{k=1}^{K} 4N_{k}f_{km}^{2}-{N\mu}_{m}^{2} \right)}{\frac{1}{M}\sum_{m=1}^{M} \frac{1}{\sigma_{m}^{2}}\sum_{k=1}^{K} \left( 2f_{km}-2f_{km}^{2} \right)}$$

$$=\frac{\sum_{m=1}^{M} \frac{1}{\sigma_{m}^{2}}\left( \sum_{k=1}^{K} 4N_{k}f_{km}^{2}-{N\mu}_{m}^{2} \right)}{\sum_{m=1}^{M} \frac{1}{\sigma_{m}^{2}}\sum_{k=1}^{K} \left( 2f_{km}-2f_{km}^{2} \right)}$$

$$<\frac{\sum_{m=1}^{M} \frac{4}{\sigma_{m}^{2}}\sum_{k=1}^{K} N_{k}f_{km}^{2}}{\sum_{m=1}^{M} \frac{2}{\sigma_{m}^{2}}\sum_{k=1}^{K} \left( f_{km}-f_{km}^{2} \right)}$$

When $f_{km}\to0$ for $k=1,2,\cdots K$ and $m=1,2,\cdots M$, we have $F_{\mathrm{PC}}\to0$.

**Text S4: Proofs of rare variants on the eigenvalues**

For submatrix $Z_{kk}$, $k=1,2, \cdots,K$, it has the largest eigenvalue $z^{k}+(N_{k}-1)z^{kk}$ with the associated eigenvector $\boldsymbol{1}_{N_{k}}/\sqrt{N_{k}}$ and additional $N_{k}-1$ eigenvalues $z^{k}-z^{kk}$ [Ma and Amos 2010], where $\boldsymbol{1}_{N_{k}}$ is the vector of dimension $N_{k}$ and with each element as 1. That is, there exist $N_{k}-1$ eigenvectors $\boldsymbol{e}_{ki}$ that satisfy $Z_{kk} \boldsymbol{e}_{ki}=(z^{k}-z^{kk})\boldsymbol{e}_{ki}$ and $\boldsymbol{1}_{N_{k}}^{T}\boldsymbol{e}_{ki}=0$, $i=1, \cdots,N_{k}-1$. By padding $\sum_{i=1}^{k-1} N_{i}$ zeros before $\boldsymbol{e}_{ki}$ and $\sum_{i=k+1}^{N} N_{i}$ zeros after it, $\boldsymbol{e}_{ki}$ can be expanded to vectors of dimension $N$. Then,

$\left( \begin{matrix} \begin{matrix} Z_{11} & Z_{12} \\ Z_{12}^{T} & Z_{22} \end{matrix} & \begin{matrix} \cdots& Z_{1K} \\ \cdots& Z_{2K} \end{matrix} \\ \begin{matrix} \vdots& \vdots\\ Z_{1K}^{T} & Z_{2K}^{T} \end{matrix} & \begin{matrix} \ddots& \vdots\\ \cdots& Z_{KK} \end{matrix} \end{matrix} \right)\left( \begin{matrix} \begin{matrix} 0 \\ \vdots\end{matrix} \\ \begin{matrix} \boldsymbol{e}_{ki} \\ \vdots\\ 0 \end{matrix} \end{matrix} \right)=\left( \begin{matrix} \begin{matrix} Z_{1k}\boldsymbol{e}_{ki} \\ \vdots\end{matrix} \\ \begin{matrix} Z_{kk}\boldsymbol{e}_{ki} \\ \vdots\\ Z_{kK}^{T}\boldsymbol{e}_{ki} \end{matrix} \end{matrix} \right)=(z^{k}-z^{kk})\left( \begin{matrix} \begin{matrix} 0 \\ \vdots\end{matrix} \\ \begin{matrix} \boldsymbol{e}_{ki} \\ \vdots\\ 0 \end{matrix} \end{matrix} \right)$.

The last equality comes from the facts that $Z_{lk}$, $l\neq k$ are of structure (9) and $\boldsymbol{e}_{ki}$ are orthogonal with $\boldsymbol{1}_{N_{k}}$. Thus, *Z* have $N_{k}-1$ eigenvalues of value $z^{k}-z^{kk}$, and the associated eigenvectors are ${[0,\cdots\boldsymbol{e}_{ki}^{T},\cdots0]}^{T}$, $i=1, \cdots,N_{k}-1$, $k=1,2, \cdots, K$. Therefore, sum of the $N-K$ eigenvalues

$$\sum_{i=K+1}^{N} \lambda_{i}=\sum_{k=1}^{K} \left( N_{k}-1 \right)\left( z^{k}-z^{kk} \right)$$

Since the trace of *Z*

$\mathrm{tr}\left( Z \right)=\sum_{k=1}^{K} N_{k}z^{k}=\sum_{i=1}^{N} \lambda_{i}$,

sum of the *K* eigenvalues

$$\sum_{k=1}^{K} \lambda_{k}=\sum_{k=1}^{K} N_{k}z^{k}-\sum_{k=1}^{K} \left( N_{k}-1 \right)\left( z^{k}-z^{kk} \right)$$

$=\sum_{k=1}^{K} N_{k}z^{kk}+\sum_{k=1}^{K} \left( z^{k}-z^{kk} \right)$. □

The *K* eigenvalues and eigenvectors can be obtained from the solving the eigen-decomposition of the following matrix [23]

$Z^{*}=\left( \begin{matrix} \begin{matrix} z^{1}-z^{11}+N_{1}z^{11} & N_{2}z^{12} \\ N_{1}z^{12} & z^{2}-z^{22}+N_{2}z^{22} \end{matrix} & \begin{matrix} \cdots& N_{K}z^{1K} \\ \cdots& N_{K}z^{2K} \end{matrix} \\ \begin{matrix} \vdots& \vdots\\ N_{1}z^{1K} & N_{2}z^{2K} \end{matrix} & \begin{matrix} \ddots& \vdots\\ \cdots& z^{K}-z^{KK}+N_{K}z^{KK} \end{matrix} \end{matrix} \right)$.

We can decompose $Z^{*}$ into two parts

$$Z^{*}=Z_{1}^{*}+Z_{2}^{*}$$

where

$Z_{1}^{*}=\left( \begin{matrix} \begin{matrix} z^{1}-z^{11} & 0 \\ 0 & z^{2}-z^{22} \end{matrix} & \begin{matrix} \cdots& 0 \\ \cdots& 0 \end{matrix} \\ \begin{matrix} \vdots& \vdots\\ 0 & 0 \end{matrix} & \begin{matrix} \ddots& \vdots\\ \cdots& z^{K}-z^{KK} \end{matrix} \end{matrix} \right)$,

$Z_{2}^{*}=\left( \begin{matrix} \begin{matrix} {N_{1}z}^{11} & N_{2}z^{12} \\ N_{1}z^{12} & {N_{2}z}^{22} \end{matrix} & \begin{matrix} \cdots& N_{K}z^{1K} \\ \cdots& N_{K}z^{2K} \end{matrix} \\ \begin{matrix} \vdots& \vdots\\ N_{1}z^{1K} & N_{2}z^{2K} \end{matrix} & \begin{matrix} \ddots& \vdots\\ \cdots& N_{K}z^{KK} \end{matrix} \end{matrix} \right)$.

Since

$$\boldsymbol{1}_{K}^{T}\left( \begin{matrix} \begin{matrix} N_{1} & 0 \\ 0 & N_{2} \end{matrix} & \begin{matrix} \cdots& 0 \\ \cdots& 0 \end{matrix} \\ \begin{matrix} \vdots& \vdots\\ 0 & 0 \end{matrix} & \begin{matrix} \ddots& \vdots\\ \cdots& N_{K} \end{matrix} \end{matrix} \right)Z_{2}^{*}\boldsymbol{1}_{K}=\boldsymbol{1}_{K}^{T}\left( \begin{matrix} \begin{matrix} N_{1}^{2}z^{11} & N_{1}N_{2}z^{12} \\ N_{2}N_{1}z^{12} & N_{2}^{2}z^{22} \end{matrix} & \begin{matrix} \cdots& N_{1}N_{K}z^{1K} \\ \cdots& N_{2}N_{K}z^{2K} \end{matrix} \\ \begin{matrix} \vdots& \vdots\\ N_{K}N_{1}z^{1K} & N_{K}N_{2}z^{2K} \end{matrix} & \begin{matrix} \ddots& \vdots\\ \cdots& N_{K}^{2}z^{KK} \end{matrix} \end{matrix} \right)\boldsymbol{1}_{K}$$

=$\sum_{k=1}^{K} N_{k}^{2}z^{kk}+\sum_{k=1}^{K} \sum_{\begin{aligned} l=1 \\ l\neq k \end{aligned}}^{K} N_{k}N_{l}z^{kl}$

$$=\sum_{k=1}^{K} \frac{N_{k}^{2}}{M}\sum_{m=1}^{M} \frac{\left( 2f_{km}-\mu_{m} \right)^{2}}{\sigma_{m}^{2}}+\sum_{k=1}^{K} \sum_{\begin{aligned} l=1 \\ l\neq k \end{aligned}}^{K} \frac{N_{k}N_{l}}{M}\sum_{m=1}^{M} \frac{\left( 2f_{km}-\mu_{m} \right)\left( 2f_{lm}-\mu_{m} \right)}{\sigma_{m}^{2}}$$

$$=\frac{1}{M}\sum_{m=1}^{M} \sum_{k=1}^{K} \frac{{N_{k}^{2}\left( 2f_{km}-\mu_{m} \right)}^{2}}{\sigma_{m}^{2}}+\frac{1}{M}\sum_{m=1}^{M} \sum_{k=1}^{K} \sum_{\begin{aligned} l=1 \\ l\neq k \end{aligned}}^{K} \frac{N_{k}N_{l}\left( 2f_{km}-\mu_{m} \right)\left( 2f_{lm}-\mu_{m} \right)}{\sigma_{m}^{2}}$$

$$=\frac{1}{M}\sum_{m=1}^{M} \frac{\left[ \sum_{k=1}^{K} N_{k}\left( 2f_{km}-\mu_{m} \right) \right]^{2}}{\sigma_{m}^{2}}$$

$$=0$$

Therefore, $Z_{2}^{*}$ has an eigenvalue with value zero. According to the Weyl’s theorem [Lütkepohl 1997], it can be shown that the smallest eigenvalue of $Z^{*}$ is larger than the minimum of $z^{k}-z^{kk}$ and less than the maximum of $z^{k}-z^{kk}$, $k=1,2,\cdots,K$. Note that this eigenvalue is the smallest among the *K* eigenvalues containing the inter-population variance, and it is smaller than some of the other $N-K$ eigenvalues of *Z*. That is, the $K-1$ largest eigenvalues of $Z^{*}$ are usually the largest eigenvalues of *Z*. Whereas, the smallest eigenvalue of $Z^{*}$ is not the *K*-th largest eigenvalues of *Z*.

Specially, when $z^{k}-z^{kk}=\sigma^{2}$, $k=1,2,\cdots,K$, the smallest eigenvalue of $Z^{*}$ is $\sigma^{2}$ and all inter-population variance are contained in the largest $K-1$ PCs. When the total sample size is large and the portions of populations remain,

$Z^{*}\approx Z_{2}^{*}$.

In this case, any inter-population variance contained in the smallest eigenvalue is negligible, almost all information on the population structure is contained in the largest $K-1$ PCs.

For cases with two populations, eigenvalues can be obtained by solving the quadratic equation

$\det\left( \begin{matrix} z^{1}-z^{11}+N_{1}z^{11}-\lambda& N_{2}z^{12} \\ N_{1}z^{12} & z^{2}-z^{22}+N_{2}z^{22}-\lambda\end{matrix} \right)=0$,

which is,

$$\lambda^{2}-\left( N_{1}z^{11}+N_{2}z^{22}+z^{1}-z^{11}+z^{2}-z^{22} \right)\lambda+\left( N_{1}z^{11}+z^{1}-z^{11} \right)\left( N_{2}z^{22}+z^{2}-z^{22} \right)-N_{1}N_{2}\left( z^{12} \right)^{2}=0$$

Then, we have

$\lambda_{1}=\frac{N_{1}z^{11}}{2}+\frac{N_{2}z^{22}}{2}+\frac{z^{1}-z^{11}}{2}+\frac{z^{2}-z^{22}}{2}+\frac{\sqrt{a}}{2}$,

$\lambda_{2}=\frac{N_{1}z^{11}}{2}+\frac{N_{2}z^{22}}{2}+\frac{z^{1}-z^{11}}{2}+\frac{z^{2}-z^{22}}{2}-\frac{\sqrt{a}}{2}$,

where

$a=\left[ \left( z^{1}-z^{11} \right)-\left( z^{2}-z^{22} \right)+{N_{1}z}^{11}-{N_{2}z}^{22} \right]^{2}+4N_{1}N_{2}\left( z^{12} \right)^{2}$.

When $z^{1}-z^{11}=z^{2}-z^{22}=\frac{\sigma_{W}^{2}}{2}$

$$a=\left( {N_{1}z}^{11}-{N_{2}z}^{22} \right)^{2}+4N_{1}N_{2}\left( z^{12} \right)^{2}$$

$$=N_{1}^{2}\left( z^{11} \right)^{2}-2N_{1}N_{2}z^{11}z^{22}+N_{2}^{2}\left( z^{22} \right)^{2}+4N_{1}N_{2}\left( z^{12} \right)^{2}$$

$$=N_{1}^{2}\left( z^{11} \right)^{2}+2N_{1}N_{2}z^{11}z^{22}+N_{2}^{2}\left( z^{22} \right)^{2}+4N_{1}N_{2}\left[ z^{11}z^{22}-\left( z^{12} \right)^{2} \right]$$

$=\left( N_{1}z^{11}+N_{2}z^{22} \right)^{2}$.

The last equality follows by the fact that

$\left( \begin{matrix} N_{1} & 0 \\ 0 & N_{2} \end{matrix} \right)\left( \begin{matrix} z^{11} & z^{12} \\ z^{12} & z^{22} \end{matrix} \right)\left( \begin{matrix} N_{1} & 0 \\ 0 & N_{2} \end{matrix} \right)$ is of rank one, thus

$\det\left( \begin{matrix} z^{11} & z^{12} \\ z^{12} & z^{22} \end{matrix} \right)=z^{11}z^{22}-\left( z^{12} \right)^{2}=0$.

Therefore, we have

$$\lambda_{1}=N_{1}z^{11}+N_{2}z^{22}+\frac{\sigma_{W}^{2}}{2}$$

$\lambda_{2}=\frac{\sigma_{W}^{2}}{2}$.

**Text S5: Proof of equation (15)**

Suppose that $\boldsymbol{x}_{i}$, $k=1,2, \cdots,N$ are the eigenvectors associated with the eigenvalues $\lambda_{i}$, $k=1,2, \cdots,N$, respectively, of the EGRM *Z*.

$$d^{2}=\sum_{k=1}^{K} \left[ \sqrt{\lambda_{k}}\boldsymbol{x}_{k}-\left( \sqrt{\lambda_{k}}\boldsymbol{x}_{k}^{T}\boldsymbol{1}_{N}/N \right)\boldsymbol{1}_{N} \right]^{T}\left[ \sqrt{\lambda_{k}}\boldsymbol{x}_{k}-\left( \sqrt{\lambda_{k}}\boldsymbol{x}_{k}^{T}\boldsymbol{1}_{N}/N \right)\boldsymbol{1}_{N} \right]$$

$$=\sum_{k=1}^{K} \lambda_{k}\boldsymbol{x}_{k}^{T}\boldsymbol{x}_{k}-\frac{1}{N}\sum_{k=1}^{K} \lambda_{k}\boldsymbol{1}_{N}^{T}\boldsymbol{x}_{k}\boldsymbol{x}_{k}^{T}\boldsymbol{1}_{N}$$

$$=\sum_{k=1}^{K} \lambda_{k}-\frac{1}{N}\boldsymbol{1}_{N}^{T}\left( \sum_{k=1}^{K} \lambda_{k}\boldsymbol{x}_{k}\boldsymbol{x}_{k}^{T} \right)\boldsymbol{1}_{N}$$

$$=\sum_{k=1}^{K} \lambda_{k}-\frac{1}{N}\boldsymbol{1}_{N}^{T}\left( Z-\sum_{k=K+1}^{N} \lambda_{k}\boldsymbol{x}_{k}\boldsymbol{x}_{k}^{T} \right)\boldsymbol{1}_{N}$$

$$=\sum_{k=1}^{K} \lambda_{k}-\frac{1}{N}\boldsymbol{1}_{N}^{T}Z\boldsymbol{1}_{N}$$

$$=\sum_{k=1}^{K} \lambda_{k}-\frac{1}{N}\sum_{k=1}^{K} N_{k}z^{k}-\frac{1}{N}\sum_{k=1}^{K} N_{k}\left( N_{k}-1 \right)z^{kk}-\frac{1}{N}\sum_{k=1}^{K} \sum_{\begin{aligned} l=1 \\ l\neq k \end{aligned}}^{K} N_{k}N_{l}z^{kl}$$

$$=\sum_{k=1}^{K} \lambda_{k}-\frac{1}{N}\sum_{k=1}^{K} N_{k}\left( z^{k}-z^{kk} \right)-\frac{1}{N}\sum_{k=1}^{K} N_{k}^{2}z^{kk}-\frac{1}{N}\sum_{k=1}^{K} \sum_{\begin{aligned} l=1 \\ l\neq k \end{aligned}}^{K} N_{k}N_{l}z^{kl}$$

$$=\sum_{k=1}^{K} \lambda_{k}-\frac{1}{N}\sum_{k=1}^{K} N_{k}\left( z^{k}-z^{kk} \right)$$

In the fourth equality, we used the spectral decomposition $Z=\sum_{i=1}^{N} \lambda_{i}\boldsymbol{x}_{i}\boldsymbol{x}_{i}^{T}$, and the fifth equality follows by the fact that the $N-K$ eigenvectors are orthogonal with $\boldsymbol{1}_{N}$. The eighth equality follows by the identity shown in Supplementary Text S3

$\sum_{k=1}^{K} N_{k}^{2}z^{kk}+\sum_{k=1}^{K} \sum_{\begin{aligned} l=1 \\ l\neq k \end{aligned}}^{K} N_{k}N_{l}z^{kl}=0$.

**Reference**

Lütkepohl H. Handbook of Matrices. Wiley. 1997.

**Table S1. Theoretical and empirical values of the inter-population covariance of EGRM (0.4<MAF≤0.5).**

| Theoretical/ Empirical | EUR | EAS | AMR | SAS | AFR |
| --- | --- | --- | --- | --- | --- |
| EUR |  |  |  |  |  |
| EAS | -0.0512/-0.0468 |  |  |  |  |
| AMR | 0.0489/0.0447 | 0.0032/0.0029 |  |  |  |
| SAS | 0.0303/0.0277 | 0.0107/0.0098 | 0.0107/0.0098 |  |  |
| AFR | -0.1052/-0.0961 | -0.1395/-0.1275 | -0.087/-0.0795 | -0.096/-0.0878 |  |

**Table S2. Theoretical and empirical values of the inter-population covariance of EGRM (0.3<MAF≤0.4).**

| Theoretical/ Empirical | EUR | EAS | AMR | SAS | AFR |
| --- | --- | --- | --- | --- | --- |
| EUR |  |  |  |  |  |
| EAS | -0.0567/-0.0517 |  |  |  |  |
| AMR | 0.0468/0.0427 | -0.0002/-0.0002 |  |  |  |
| SAS | 0.0293/0.0267 | 0.0085/0.0077 | 0.0094/0.0086 |  |  |
| AFR | -0.0986/-0.0900 | -0.1295/-0.1182 | -0.0812/-0.0741 | -0.0931/-0.085 |  |

**Table S3. Theoretical and empirical values of the inter-population covariance of EGRM (0.2<MAF≤0.3).**

| Theoretical/ Empirical | EUR | EAS | AMR | SAS | AFR |
| --- | --- | --- | --- | --- | --- |
| EUR |  |  |  |  |  |
| EAS | -0.0548/-0.0502 |  |  |  |  |
| AMR | 0.0441/0.0403 | -0.0013/-0.0012 |  |  |  |
| SAS | 0.0270/0.0247 | 0.0072/0.0066 | 0.0090/0.0082 |  |  |
| AFR | -0.0930/-0.0851 | -0.1215/-0.1112 | -0.0763/-0.0698 | -0.0874/-0.0800 |  |

**Table S4. Theoretical and empirical values of the inter-population covariance of EGRM (0.1<MAF≤0.2).**

| Theoretical/ Empirical | EUR | EAS | AMR | SAS | AFR |
| --- | --- | --- | --- | --- | --- |
| EUR |  |  |  |  |  |
| EAS | -0.0417/-0.0383 |  |  |  |  |
| AMR | 0.0408/0.0374 | 0.0017/0.0015 |  |  |  |
| SAS | 0.0292/0.0269 | 0.0119/0.0109 | 0.0106/0.0097 |  |  |
| AFR | -0.0945/-0.0868 | -0.1187/-0.109 | -0.0745/-0.0685 | -0.0928/-0.0852 |  |

**Table S5. Theoretical and empirical values of the inter-population covariance of EGRM (0.01<MAF≤0.1).**

| Theoretical/ Empirical | EUR | EAS | AMR | SAS | AFR | |
| --- | --- | --- | --- | --- | --- | --- |
| EUR |  |  |  |  |  | |
| EAS | 0.0034/0.0032 |  |  |  |  | |
| AMR | 0.0242/0.0228 | 0.0092/0.0087 |  |  |  | |
| SAS | 0.0241/0.0227 | 0.0188/0.0177 | 0.0110/0.0104 |  |  | |
| AFR | -0.0837/-0.0789 | -0.0885/-0.0835 | -0.0601/-0.0566 | -0.0822/-0.0775 | |  |

**Table S6. Theoretical and empirical values of the inter-population covariance of EGRM (0.0001<MAF≤0.01).**

| Theoretical/ Empirical | EUR | EAS | AMR | SAS | AFR |
| --- | --- | --- | --- | --- | --- |
| EUR |  |  |  |  |  |
| EAS | -0.0004/-0.0004 |  |  |  |  |
| AMR | 0.0006/0.0006 | -0.0005/-0.0005 |  |  |  |
| SAS | -0.0001/-0.0001 | -0.0006/-0.0006 | -0.0005/-0.0005 |  |  |
| AFR | -0.0032/-0.0031 | -0.0034/-0.0034 | -0.0022/-0.0022 | -0.0036/-0.0036 |  |

**Table S7. MAFs of the six SNPs used in the simulations.**

| MAF Bin | SNP | $f_{\mathrm{Total}}$ | $f_{\mathrm{EUR}}$ | $f_{\mathrm{EAS}}$ | $f_{\mathrm{AMR}}$ | $f_{\mathrm{SAS}}$ | $f_{\mathrm{AFR}}$ |
| --- | --- | --- | --- | --- | --- | --- | --- |
| 1 | rs1000312 | 0.4433 | 0.337 | 0.4603 | 0.4597 | 0.6135 | 0.3767 |
| 2 | rs1000073 | 0.3662 | 0.5835 | 0.1776 | 0.3977 | 0.2587 | 0.4077 |
| 3 | rs1000348 | 0.279 | 0.4205 | 0.2054 | 0.3098 | 0.228 | 0.2489 |
| 4 | rs1000282 | 0.1937 | 0.2078 | 0.2212 | 0.2046 | 0.1656 | 0.177 |
| 5 | rs1000315 | 0.03395 | 0.06759 | 0 | 0.04179 | 0.06953 | 0.003782 |
| 6 | rs10081977 | 0.004792 | 0 | 0 | 0.001441 | 0 | 0.0174 |

**Table S8. Expected variance and covariance with and without the knowledge of $\boldsymbol{\mu}_{\boldsymbol{m}}$ and $\boldsymbol{\sigma}_{\boldsymbol{m}}$.**

|  | SNP | EUR | EAS | AMR | SAS | AFR |
| --- | --- | --- | --- | --- | --- | --- |
| $z^{k}$ | rs1000312 | 1.00/1.00 | 0.97**/**0.97 | 1.04**/**1.04 | 1.18/1.18 | 0.95/0.95 |
|  | rs1000073 | 1.57/1.57 | 0.90/0.91 | 1.04/1.04 | 0.89/0.89 | 1.07/1.07 |
|  | rs1000348 | 1.41/1.41 | 0.89/0.89 | 1.06/1.06 | 0.90/0.90 | 0.92/0.92 |
|  | rs1000282 | 1.08/1.08 | 1.05/1.06 | 1.06/1.06 | 0.89/0.90 | 0.90/0.90 |
|  | rs1000315 | 1.90/1.90 | 0.08/0.08 | 1.39/1.39 | 2.31/2.33 | 0.25/0.24 |
|  | rs10081977 | 0.01/0.01 | 0.01/0.01 | 0.27/0.31 | 0.01/0.01 | 4.86/5.18 |
| $z^{kk}$ | rs1000312 | 0.09/0.10 | 0.02/0.02 | -0.02/-0.02 | 0.18/0.18 | 0.01/0.01 |
|  | rs1000073 | 0.49/0.49 | 0.31/0.31 | -0.01/-0.01 | 0.08/0.08 | 0.00/0.00 |
|  | rs1000348 | 0.14/0.14 | 0.02/0.02 | 0.00/0.00 | 0.00/0.00 | -0.02/-0.02 |
|  | rs1000282 | 0.01/0.01 | 0.02/0.02 | -0.02/-0.02 | -0.04/-0.04 | 0.01/0.01 |
|  | rs1000315 | 0.11/0.10 | 0.08/0.08 | 0.00/0.00 | 0.07/0.07 | 0.05/0.05 |
|  | rs10081977 | 0.01/0.01 | 0.01/0.01 | 0.00/0.00 | 0.01/0.01 | 0.07/0.05 |

The first values are theoretical values assuming the knowledge of $\mu_{m}$ and $\sigma_{m}$; and the second values are empirical values using the estimates of $\mu_{m}$ and $\sigma_{m}$.
